# Long-term clinical outcome of atrial fibrillation ablation in patients with history of mitral valve surgery

**DOI:** 10.3389/fcvm.2022.928974

**Published:** 2022-12-21

**Authors:** Alexandre Almorad, Louisa O'Neill, Jean-Yves Wielandts, Kris Gillis, Benjamin De Becker, Yosuke Nakatani, Carlo De Asmundis, Saverio Iacopino, Thomas Pambrun, La Meir Marc, Pierre Jaïs, Michel Haïssaguerre, Mattias Duytschaever, Jean-Baptista Chierchia, Nicolas Derval, Sébastien Knecht

**Affiliations:** ^1^Department of Cardiology, AZ Sint Jan Hospital Bruges, Bruges, Belgium; ^2^Heart Rhythm Management Centre, Postgraduate Program in Cardiac Electrophysiology and Pacing, European Reference Networks Guard-Heart, Universitair Ziekenhuis Brussel - Vrije Universiteit Brussel, Brussels, Belgium; ^3^Department of Cardiac Pacing and Electrophysiology, Hospital Cardiologique du Haut-Lévêque, CHU de Bordeaux, Avenue de Magellan, Pessac, France

**Keywords:** atrial fibrillation, mitral valve surgery, ablation, atrial tachyarrhythmias, antiarrhythmic drugs

## Abstract

**Aims:**

Atrial fibrillation (AF) occurs frequently after mitral valve (MV) surgery. This study aims to evaluate the efficacy and long-term clinical outcomes after the first AF ablation in patients with prior MV surgery.

**Methods:**

Sixty consecutive patients with a history of MV surgery without MAZE referred to three European centers for a first AF ablation between 2007 and 2017 (group 1) were retrospectively enrolled. They were matched (propensity score match) with 60 patients referred for AF ablation without prior MV surgery (group 2).

**Results:**

After the index ablation, 19 patients (31.7%) from group 1 and 24 (40%) from group 2 had no recurrence of atrial arrhythmias (ATa) (*p* = 0.3). After 62 (48–84) months of follow-up and 2 (2–2) procedures, 90.0% of group 1 and 95.0% of group 2 patients were in sinus rhythm (*p* = 0.49). In group 1, 19 (31.7%) patients had mitral stenosis, and 41 (68.3%) had mitral regurgitation. Twenty-seven (45.0%) patients underwent mechanical valve replacement and 33 (55.0%) MV annuloplasty. At the final follow-up, 28 (46.7%) and 33 (55.0%) patients were off antiarrhythmic drugs (*p* = 0.46). ATa recurrence was seen more commonly in patients with prior MV surgery (54 vs. 22%, respectively, *p* < 0.05). No major complication occurred.

**Conclusion:**

Long-term freedom of atrial arrhythmias after atrial fibrillation catheter ablation is achievable and safe in patients with a history of mitral valve surgery. In AF patients without a history of mitral valve surgery, repeated procedures are needed to maintain sinus rhythm.

## Introduction

Atrial tachyarrhythmias (ATa) are a common cause of morbidity in patients with mitral valve disease. Left atrial volume and pressure loading in the setting of stenotic and regurgitant mitral valvular disease results in atrial electrical and structural remodeling predisposing to atrial arrhythmogenesis (1). Although surgical correction is associated with an improvement in hemodynamics and reduction in left atrial (LA) dimensions (2), the risk of post-surgical ATa remains elevated (1–3) and is associated with increased mortality and morbidity (4, 5). As well as ongoing pre-existing arrhythmia (3) *de novo* atrial fibrillation (AF) is associated with increased age and LA size while reentry mechanisms can arise from sites of surgical incision and scarring (6). Catheter ablation can be challenging in this cohort, given the degree of intrinsic arrhythmogenic remodeling and the presence of surgical incisions and scars. Nevertheless, symptom burden is often high in this population and ablation may offer a significant quality of life benefit to the patient (7, 8).

To the best of our knowledge, little data exist on long-term efficacy, beyond 2 years, of AF catheter ablation after MV surgery. The aim of this study is, therefore, to evaluate long-term safety and efficacy outcomes of catheter ablation in this cohort.

## Methods

### Study population

An electronic medical database of three Europeans was screened for patients with a history of successful surgical correction of mitral stenosis or regurgitation and without concomitant MAZE surgery, referred for a first AF ablation from January 2008 to December 2017 across three European centers, were screened for inclusion (group 1). Patients with no or mild residual mitral regurgitation and with both paroxysmal and persistent AF were included. Those with a history of prior surgical or percutaneous ATa ablation or congenital cardiomyopathy were excluded. After collecting patients' written informed consent, a detailed case report form including clinical and procedural characteristics and follow-up was filled and incorporated into a common database shared by the three centers.

A comparison was made to a group of patients, matched for age, gender, body mass index, follow-up duration, and AF type with a history of AF ablation and no prior MV surgery (group 2).

### Radiofrequency catheter ablation

Procedures were carried out under local or general anesthesia depending on the institution. Ablation strategy was according to operator discretion and ranged from pulmonary vein isolation (PVI) only to more extensive strategies including linear ablation at the LA roof and mitral isthmus, ablation of complex fractionated atrial electrograms, cavo-tricuspid isthmus ablation and superior vena cava isolation, depending on AF duration and persistence. The PVI-only strategy was performed either with a radiofrequency catheter or a single-shot cryoballoon.

In the case of repeat procedures for recurrent ATa, persistent isolation of the pulmonary veins and block across lines (if applicable) were evaluated with RF ablation performed, where necessary, to achieve re-isolation or block. Further ablation was eventually performed according to the operator's discretion. Electrical cardioversion was performed at the end of the procedure in the event of failure to restore sinus rhythm.

### Follow-up

All patients underwent a clinical evaluation and a 12-lead electrocardiogram (ECG) at 1, 3, 6, and 12 months as well as a yearly 24-h Holter. ATa recurrence was defined according to the HRS/EHRA/ECAS expert consensus document as any recurrence of atrial arrhythmia >30 s (9) and a 3-month blanking period was applied.

Complications including vascular damage, thromboembolism, pericardial effusion, esophageal fistula, mechanical valve damage, atrioventricular block, and procedure-related death were systematically recorded.

### Statistical analysis

Statistical analyses were performed in SPSS Statistics 24 (IBM Corporation, Armonk, New York, USA). Propensity-score matching with a 1:1 ratio, without replacement, and with the nearest neighbor technique was used to create groups of patients with similar characteristics out of a database of 180 patients (age, gender, body mass index, LA volume, follow-up duration, and AF type) to compare the outcomes in patients with vs. without MV surgery undergoing ablation.

Comparison of means between groups was performed using independent samples *t*-test for normally distributed data and Mann–Whitney *U*-test for non-uniformly distributed data. Continuous variables are expressed as mean ± SD if normally distributed, medians with first and third quartiles (Q1–Q3) if non-normally distributed, and dichotomous variables as percentages were compared using the χ^2^ test. Kaplan–Meier plots were used to report arrhythmia-free survival curves for each group, and a time-to-event analysis was performed using the log-rank test. A bilateral *p*-value < 0.05 was considered statistically significant.

## Results

### Patients' and procedural characteristics

The baseline clinical characteristics of the study cohort are presented in Table 1. Sixty patients with prior MV surgery and a first AF ablation (Group 1; 65.5 ± 5.8 years, 50% women) and 60 matched patients (Group 2; 64.3 ± 6.9 years, 55% women) were studied. Patients' characteristics in both groups are summarized in Table 1.

**Table 1:** Clinical and index procedure characteristics (*n* = 120).

	**Group 1 (*n =* 60)**	**Group 2 (control, *n =* 60)**	***P*-value**
Female, *n* (%)	30 (50.0%)	33 (55.0%)	0.84
Age, mean ± SD yrs	65.5 ± 5.8	64.3 ± 6.9	0.29
BMI, mean ± SD kg/m^2^	24.9 ± 4.1	25.5 ± 4.1	0.31
Mitral regurgitation, *n* (%)	41 (68.3%)	NA	NA
Mitral stenosis, *n* (%)	19 (31.7%)	NA	NA
Mitral valve repair, *n* (%)	33 (55.0%)	NA	NA
Mitral valve replacement, *n* (%)	27 (45.0%)	NA	NA
Mechanical valve, *n* (%)	27 (45%)	NA	NA
**Type of atrial fibrillation**			
Paroxysmal AF, *n* (%)	32 (53.3%)	30 (50.0%)	0.85
Non paroxysmal AF, *n* (%)	28 (46.7%)	30 (50.0%)	0.85
Time from first AF episode, months	35.8 ± 6.5	33.0 ± 8.4	0.16
CHA_2_DS_2_-VASc Score	2,4 ± 1.4	2.2 ± 1.1	0.92
Arterial Hypertension, *n* (%)	36 (60.0%)	39 (65.0%)	0.78
Diabetes, *n* (%)	5 (7.1%)	4 (6.7%)	0.66
Congestive heart failure, *n* (%)	12 (20.0%)	7 (11.7%)	0.32
History of stroke, *n* (%)	6 (10.0%)	4 (6.7%)	0.53
Anti-arrhythmic drug before the procedure, *n* (%)	33 (55.0%)	30 (50.0%)	0.84
Betablockers, *n* (%)	25 (*41.7%*)	36 (*60.0%*)	0.07
Flecainide, *n* (%)	25 (*41.7%*)	21 (*35.0%*)	0.57
Sotalol, *n* (%)	4 (*6.7%*)	3 (*5.0%*)	0.46
Amiodarone, *n* (%)	10 (16.7%)	5 (*7.1%*)	0.27
Direct anticoagulant, *n* (%)	37 (61.7%)	40 (66.7%)	0.71
Antivitamin K, *n* (%)	34 (56.7%)	7 (11.7%)	*0.001*
LVEF, mean ± SD %	51.8 ± 7.5	52.7 ± 3.9	0.21
Left atrial diameter	47.2 ± 6.7	44.3 ± 5.5	0.08

BMI, body mass index; AF, atrial fibrillation; LVEF, left ventricular ejection fraction.

In group 1, 19 (31.7%) patients had mitral stenosis and 41 (68.3%) had mitral regurgitation. Twenty-seven (45.0%) patients underwent MV replacement with mechanical valves and 33 (55.0%) MV annuloplasty. Eighteen patients underwent concomitant surgical procedures, including coronary bypass in six patients, aortic valve replacement in three patients, tricuspid valve repair in eight patients, and interatrial closure in one patient. Surgical atrial access was granted through the right atrium and the interatrial septum in 32 patients, 12 patients through the left atrium, and 16 patients through the roof.

Procedural characteristics are shown in Table 2. Substrate ablation was performed in a higher proportion of patients in group 1 (Table 2). Procedural times (*p* = 0.47) and fluoroscopy times were similar between groups (*p* = 0.72).

**Table 2:** Procedural characteristics (*n* = 120).

	**Group 1 (*n =* 60)**	**Group 2 (control, *n =* 60)**	***P*-value**
Sinus rhythm at index procedure, *n* (%)	27 (45.0%)	43 (71.7%)	*0.003*
PVI only at index, *n* (%)	13 (21.7%)	29 (48.3%)	*0,004*
**Substrate ablation at index**			
Left atrium substrate	47	31	*0,003*
Right atrium substrate	7	1	*0,001*
Superior Vena Cava Isolation	7	1	*0,001*
Cavotricuspid Isthmus	11	2	*0,001*
Index procedure time, mean ± SD minutes	137.45 ± 30.2	134.45 ± 19.7	0.47
Index fluoroscopic time, mean ± SD minutes	8.1 ± 5.2	7.9 ± 3.9	0.72

PVI, Pulmonary vein isolation.

### Procedural outcomes and recurrence characteristics

**Figure 1:**
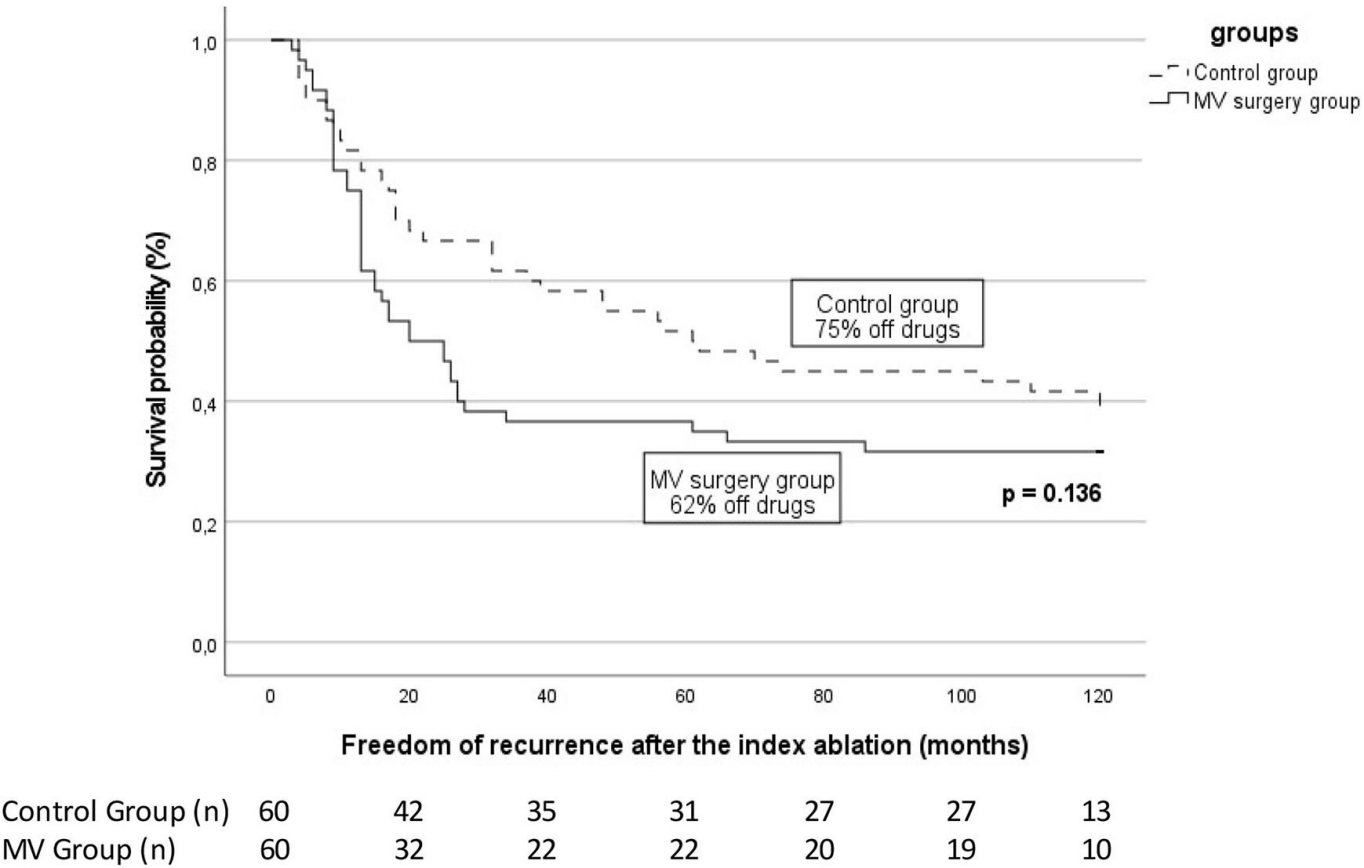
Arrhythmia-free survival after the index catheter ablation for atrial fibrillation in patients with previous mitral valve (MV) surgery patients vs. control. The proportion of patients off antiarrhythmic drugs is specified for each group. The number of patients at risk at each time interval is shown below the figure. The *p*-value reflects the log-rank significance at the end of the follow-up.

Following the index ablation, 19 patients (31.6%) from group 1 and 24 patients (40.0%) from group 2 had no recurrence of ATa (*p* = 0.34). Of these, 9 (15%) and 18 (30%) respectively were not taking antiarrhythmic drugs (AADs) (*p* = 0.22, Table 3). In those with recurrent arrhythmia, the median time to the first recurrence was similar between groups [Group 1, 13 (9–17) months vs. Group 2, 19 (8–22) months] (*p* = 0.06; Figure 1). One-, 2-, and 5-years freedom of recurrence are also shown on Kaplan–Meier curves (Supplementary material).

**Table 3:** Follow-up characteristics (*n* = 120).

	**Group 1 (*n =* 60)**	**Group 2 (control, *n =* 60)**	***P*-value**
Free of arrhythmia after index procedure, *n* (%)	19 (31.7%)	24 (40.0%)	0.34
Free of arrhythmia and off-AAD after index procedure, *n* (%)	9 (15%)	18 (30%)	0.08
Free of arrhythmia after one repeat procedure, *n* (%)	48 (80.0%)	52 (86.7%)	0.46
Free of arrhythmia at end of follow-up, *n* (%)	54 (90.0%)	57 (95.0%)	0.49
Out of AAD after last procedure, *n* (%)	28 (46.6%)	33 (55.0%)	0.46
Without any recurrence, *n* (%)	9 (*32.1*%)	18 (*54.5*%)	0.22
Number of procedures per patient, median (Q1–Q3)	2 (2–2)	2 (2-2)	0.11
Atrial tachyarrhythmias recurrence, *n* (%)	41 (68.3%)	36 (60.0%)	0.45
Atrial fibrillation, *n* (%)	19 (*46.0%*)	28 (*78.0%*)	0.09
Atrial tachycardia	22 (*54.0%*)	8 (*22.0%*)	***0.03***
Time to first recurrence, median (Q1–Q3), *n* (%)	13 (9–17)	19 (8–22)	0.06
Major complication	0	0	0.91

AAD, Antiarrhythmic drugs.

As pointed out in Table 3, 41 patients (68.3%) from group 1 and 36 patients (60.0%) from group 2 experienced recurrence. Among them, AT was more observed in group 1 than in group 2 (22 vs. 8, respectively, *p* = 0.03), whereas AF was evenly observed in both groups (19 vs. 28; *p* = 0.09).

Each patient underwent a median of 2 (2–2) ablation procedures and was followed for 62 (48–84) months. No difference was observed in both groups regarding the outcome with 54 patients (90.0%) in sinus rhythm in group 1 vs. 57 (95.0%) in group 2 (*p* = 0.49; Table 3). Twenty-eight (46.7%) and 33 (55.0%) patients were off AADs at the final follow-up in groups 1 and 2, respectively (*p* = 0.46).

No statistical difference was seen between mitral stenosis or regurgitation in terms of overall ATa recurrence, the number of procedures, and the type of ATa.

### Complications

No major peri or postoperative complications are reported. One pseudo-aneurysm and two groin hematomas were observed in groups 1 and 2 groin hematomas in group 2. Of note, mitral valve entrapment was not observed in either group.

## Discussion

In the present study, we evaluated the safety and long-term efficacy of AF catheter ablation after MV surgery in 60 patients across three experienced European centers. Our results highlight the following key findings: Catheter ablation for AF in patients with a history of MV surgery; (1) offers meaningful results with repeat procedures, similar to those seen in a matched population; (2) results in a higher rate of AT recurrence compared to control patients; and (3) has a favorable safety profile.

### Arrhythmia recurrence

This study represents the longest follow-up study of its nature in patients with prior MV surgery, without concomitant surgical ablation, referred for first-time catheter ablation for AF.

After one ablation procedure, arrhythmia-free survival is modest in our cohort, with the majority of patients experiencing a recurrence of an ATa. Similar outcomes were seen in the control group; however, these results are also reflected by other studies of patients with prior MV surgery (Table 4). In a recent study by Chen et al., only 33% of patients with prior MV replacement were arrhythmia-free at 42.7 ± 17.3 months post-ablation (10). Similarly, Hussein et al. (8) describe an Ata-free rate of 44.2% over 24 months after index ablation (8). Better results were reported by Mountantonakis et al. (11) with 71% of patients arrhythmia-free in a similar population, however, the follow-up duration was significantly shorter at only 7 ± 4 months (11) (Table 4).

**Table 4:** Clinical characteristics and outcome of ablation from studies on populations with previous mitral valve surgery.

	**Year**	**Number of patients (*n*)**	**Previous surgical ablation (*n*)**	**Follow-up (months)**	**Free of arrhythmia at end of follow-up**	**Off AAD (%)**	**Repeat procedure (%)**	**Recurrence after the index catheter ablatio*n* (%)**	**AT recurrence (%)**
Enriquez et al. (6)	2017	67	33	12	62%	NA	42%	NA	NA
Santangeli et al. (7)	2012	178	NA	11.5 ± 8.6	NA	NA	NA	36%	NA
Hussein et al. (8)	2011	81	0	24	82.7%	69%	36%	NA	NA
Chen et al. (10)	2013	21	0	42.7 ± 17.3	33%	NA	43%	67%	43%
Mountantonakis et al. (11)	2011	21	12	7 ± 4	71%	43%	43%	43%	28%
Lang et al. (12)	2005	26	0	10	73%	NA	34%	50%	23%
Lakkireddy et al. (13)	2011	50	0	12	80%	NA	34%	NA	NA

**Figure 2:**
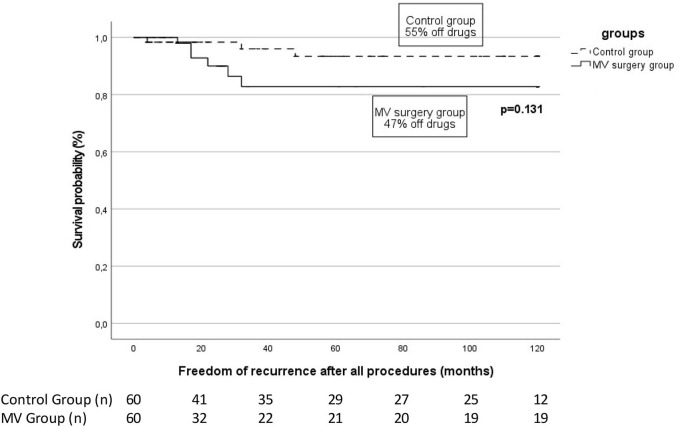
Arrhythmia-free survival in patients with previous mitral valve (MV) surgery patients vs. control at the end of follow-up after a median of 2 (2–2) catheter ablation. The proportion of patients off antiarrhythmic drugs is specified for each group. The number of patients at risk at each time interval is shown below the figure. The *p*-value reflects the Log-rank significance at the end of the follow-up.

While a single procedure appears to be insufficient to maintain sinus rhythm in these patients, after one repeat RF procedure, the rate of ATa freedom in our MV cohort increased to 80% (Figure 2) with 68.3% (41/60) of them undergoing a second procedure. Supporting these findings, high rates of repeat procedures have been described in similar population groups (6, 8).

Furthermore, at a median final follow-up of 62 (48–84) months, the long-term rate of sinus rhythm was high at 90%, with no difference seen with respect to the control group. These results suggest that, with repeat ablation, long-term outcomes similar to a control population are achievable in patients with prior MV surgery.

With regards to medium to long-term follow-up, conflicting reports exist in the literature, on smaller cohorts and/or shorter follow-ups (8, 12, 13). In a 2020 meta-analysis of 227 patients by Marazzato et al. (14) freedom from ATa at the end of follow-up was more modest at 49% after at least one repeat procedure with a significant decrease in arrhythmia-free survival seen after 2 years (14). While several studies in this meta-analysis included follow-up beyond 2 years, it also included studies of prior surgical ablation and those referred primarily for atrial tachycardia ablation, however, rendering direct comparison with the patients studied here difficult. Differences in reported post-ablation success rates in those with prior MV surgery may be explained by multiple factors including varying follow-up duration, multiple valve surgery, ablation technique, and post-ablation monitoring. Indeed, significant heterogeneity exists between the studies included in the meta-analysis mentioned above. The more favorable results seen at long-term follow-up in our study may reflect advances in current ablation and mapping technology.

At the end of the follow-up period, approximately 50% of patients in each group were off AADs. These ratios are comparable to previously published data from Mountantonakis et al. (8) and Hussein et al. (11) with patients off drugs at 43 and 69.1%, respectively (8, 11). Furthermore, without AADs, only a small proportion of the patients studied here remained free from any recurrence of atrial arrhythmia throughout follow-up. This highlights the complementary role of AADs and repeats ablation in the long-term maintenance of sinus rhythm in this patient cohort.

### Recurrence mechanisms

We report a higher rate of atrial tachycardia (AT) recurrence in the patients who underwent surgery in our cohort. This phenomenon could be explained by several mechanisms. On the one hand, valve surgery itself can contribute to slow conduction zones facilitating the appearance of re-entry circuits (15) with the type of surgical incision previously described as predictive of the development of atrial tachycardia on follow-up (16). In addition, it is well established that multiple valve surgery and coronary bypass patients, as is the case in our cohort, are associated with a higher incidence of arrhythmia (15, 16). Atrial scar and fibrosis, slow conduction zones, or incomplete lines of previous ablation procedures may also play a role in the development of AT recurrences (6). While right atrial macro re-entry circuits appear to predominate in patients undergoing first-time ablation for AT post-MV surgery, LA ATs have been more commonly described in those who have undergone concomitant surgical ablation, and become more frequent after the index catheter ablation procedure (6). Accordingly, previous studies report that the predominant mechanism for AT recurrence in patients post-MV surgery was macro re-entry in 75–99%, mostly originating from the LA in 63–100% (14, 17, 18). These observations are comparable to ours and highlight the role of LA substrate in ATa recurrence after index catheter ablation in patients with a history of MV surgery. In contrast, AF recurrences in this population may relate less to the prior surgical procedure and rather reflect the progression of an advanced atrial arrhythmia substrate secondary to the hemodynamic consequences of the valvular lesion. This point is highlighted by the authors of the 2020 meta-analysis outlined above as a probable explanation for the relationship between AF recurrences and follow-up duration (14). The high rate of sinus rhythm at the end of follow-up in this study may underscore the importance of adjunct atrial substrate ablation in this population.

### Safety

Prior safety concerns regarding catheter ablation of atrial arrhythmia in patients post-MV surgery surround the risk of damage to the prosthetic valve (19, 20) and thromboembolic events (7, 13). In this study, we report no major complications relating to ablation. In patients with a history of MV surgery and in particular with valve prostheses, catheter maneuvering may be challenging due to the presence of atrial remodeling, scarring, and the prosthesis itself. This is reflected in frequently reported increased procedural as was the case in our study. Nevertheless, with attention, the risk of valve damage appears to be low as we report no mechanical valve entrapment or post-procedural malfunction. In addition, the maintenance of periprocedural therapeutic warfarin has been reported to mitigate the increased risk of thromboembolic events (7, 13). Our findings are supported by several studies emphasizing the safety of catheter ablation in this cohort (8, 11, 14) with no difference in complication rates between those with prior MV surgery and matched controls. While Lang et al. (12) reported more procedure-related complications among patients with MV prostheses, these did not include valvular damage or thromboembolism (12).

### Clinical implications

Our study suggests that patients with symptomatic AF and a history of severe mitral valve disease requiring valvular surgery derive a potential long-term benefit from catheter ablation. Despite the need for repeat ablation procedures in most patients and the continuation of AADs in approximately half of our cohort, the results seen here were similar to a group of matched control patients, suggesting that this treatment strategy is of similar value in both cohorts. The ongoing use of AADs appears to be complimentary to repeat ablation in maintaining sinus rhythm and freedom from symptoms, and the continuation of these should not be viewed as a treatment failure in this group.

### Limitations

This is a non-randomized retrospective observational study with a sample of heterogeneous patients, including the type of MV surgery (repair or replacement) but also the physiopathology of the MV disease itself: rheumastismal and degenerative MV regurgitation propensity scores are performed to reduce the heterogeneity bias and improve the power of the analysis in retrospective studies. Nevertheless, this statistical technique is limited by nature, as only few factors can be matched, and the analysis depends on the available database. Thus, the *p*-value in Kaplan–Meier curves can be only a reflection of the low power of the study. Also, ATa recurrences were assessed by ECG rather than continuous monitoring, thus, the overall success rate may have been overestimated. Moreover, performing ablation on an operated heart, especially on the MV, is challenging and could lead to an incomplete ablation and, thus, considered a cause of the recurrence of ATa. Furthermore, due to its retrospective design, no quality of life assessment was performed during this study preventing to draw off any conclusion regarding the symptoms. In the index procedure, the PVI-only rate between groups is different, this could have impacted the final result regarding the ATa freedom, but this parameter is comparable between groups at the end of the follow-up. Whether the PVI-only strategy plays a role in the type of recurrence (AF vs. AT) is highly speculative as the number of patients would be limited to draw a powerful conclusion. In addition, over the long period covered by this study, a lot of changes in the guidelines and improvements occurred not only from a technological point of view (3D map, ContactForce, irrigation, and power control) but also technically (Ablation Index and Close protocol), this could have impacted the results of ATa freedom on the long run. Finally, this study was performed in experienced centers with strict patient selection making the results entrusted exclusively to experienced teams.

## Conclusion

In this long-term follow-up study, freedom from atrial arrhythmias after catheter ablation for atrial fibrillation is achievable and safe in patients with a history of mitral valve surgery. With repeated procedures and the use of antiarrhythmic drugs, high rates of sinus rhythm can be achieved in the long term, emphasizing the value of this treatment strategy in this cohort.

## Data availability statement

The raw data supporting the conclusions of this article will be made available by the authors, without undue reservation.

## Ethics statement

The studies involving human participants were reviewed and approved by Univeristeit Ziekenhuis Brussel Ethics Committee. The patients/participants provided their written informed consent to participate in this study.

## Author contributions

All authors listed have made a substantial, direct, and intellectual contribution to the work and approved it for publication.
