# Supplementary material for: Long-term clinical outcome of atrial fibrillation ablation in patients with history of mitral valve surgery
Source: Front Cardiovasc Med. 2022 Dec 21;9:928974. doi: 10.3389/fcvm.2022.928974 (PMC9811118; doi:10.3389/fcvm.2022.928974)
Supplement: Supplementary file 1 [file Data_Sheet_1.docx]

Supplemental Materials

Kaplan Meyer curves representing freedom of recurrence of MV patients vs Control at 1, 2 and 5 years


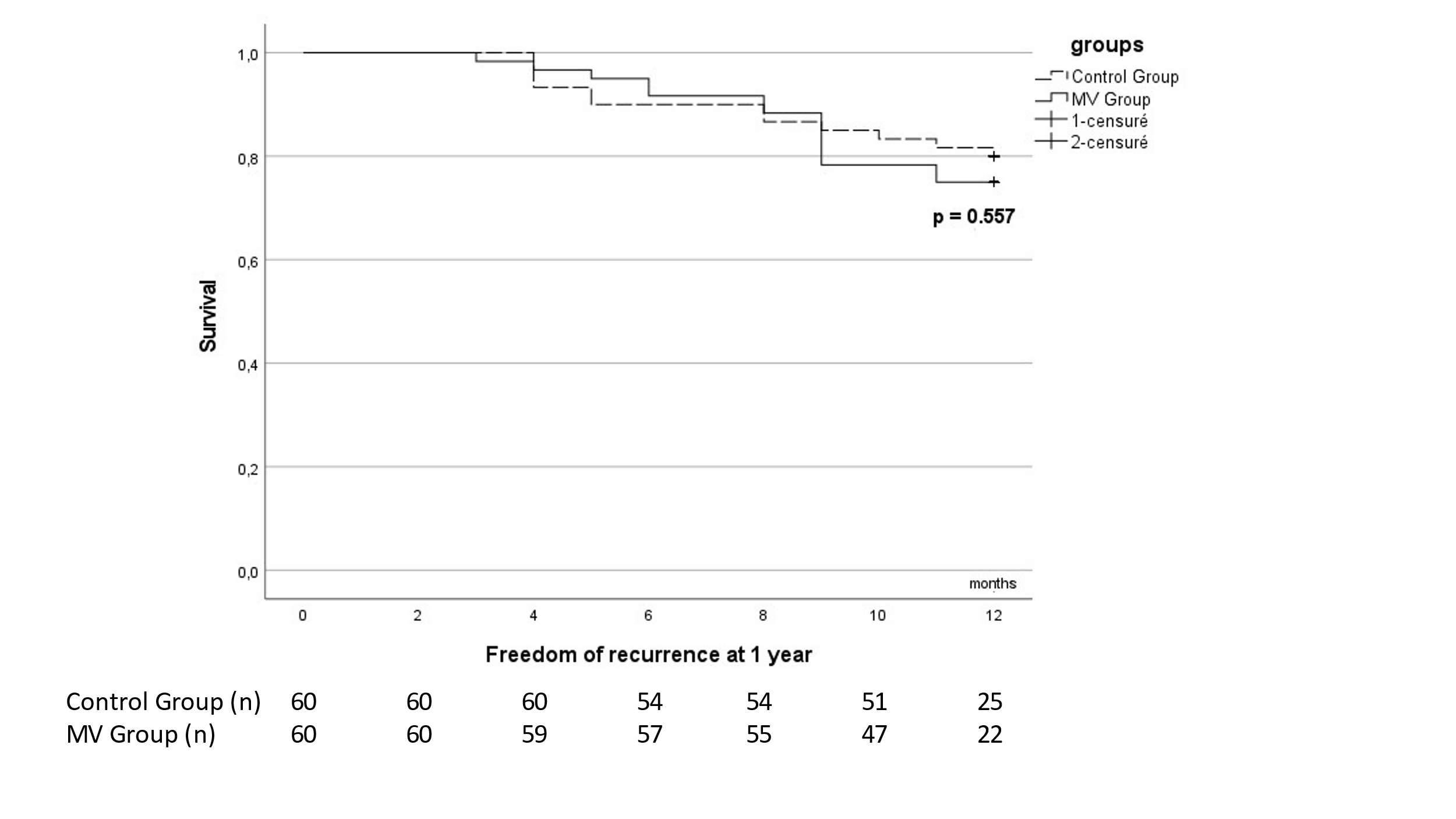


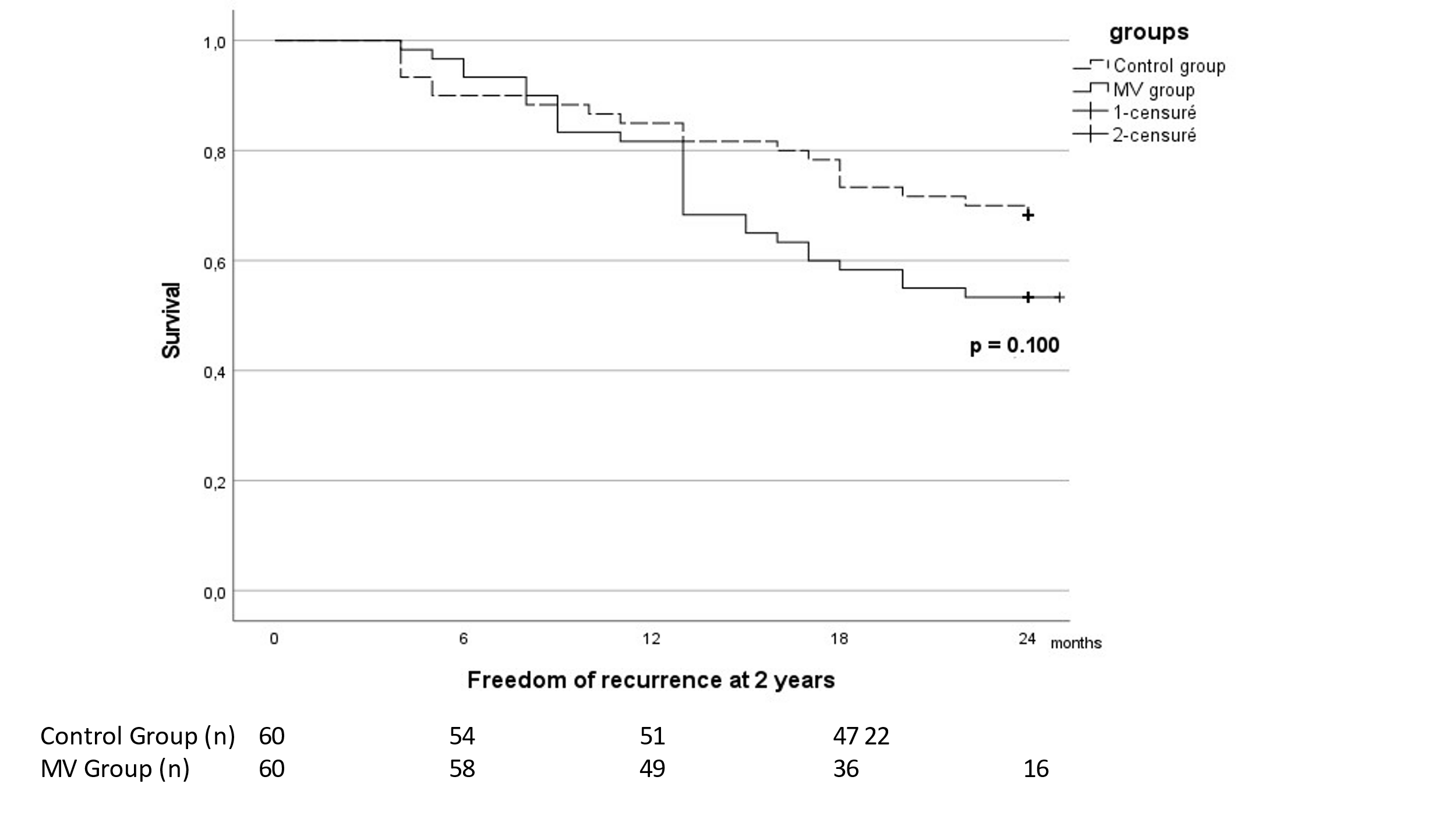


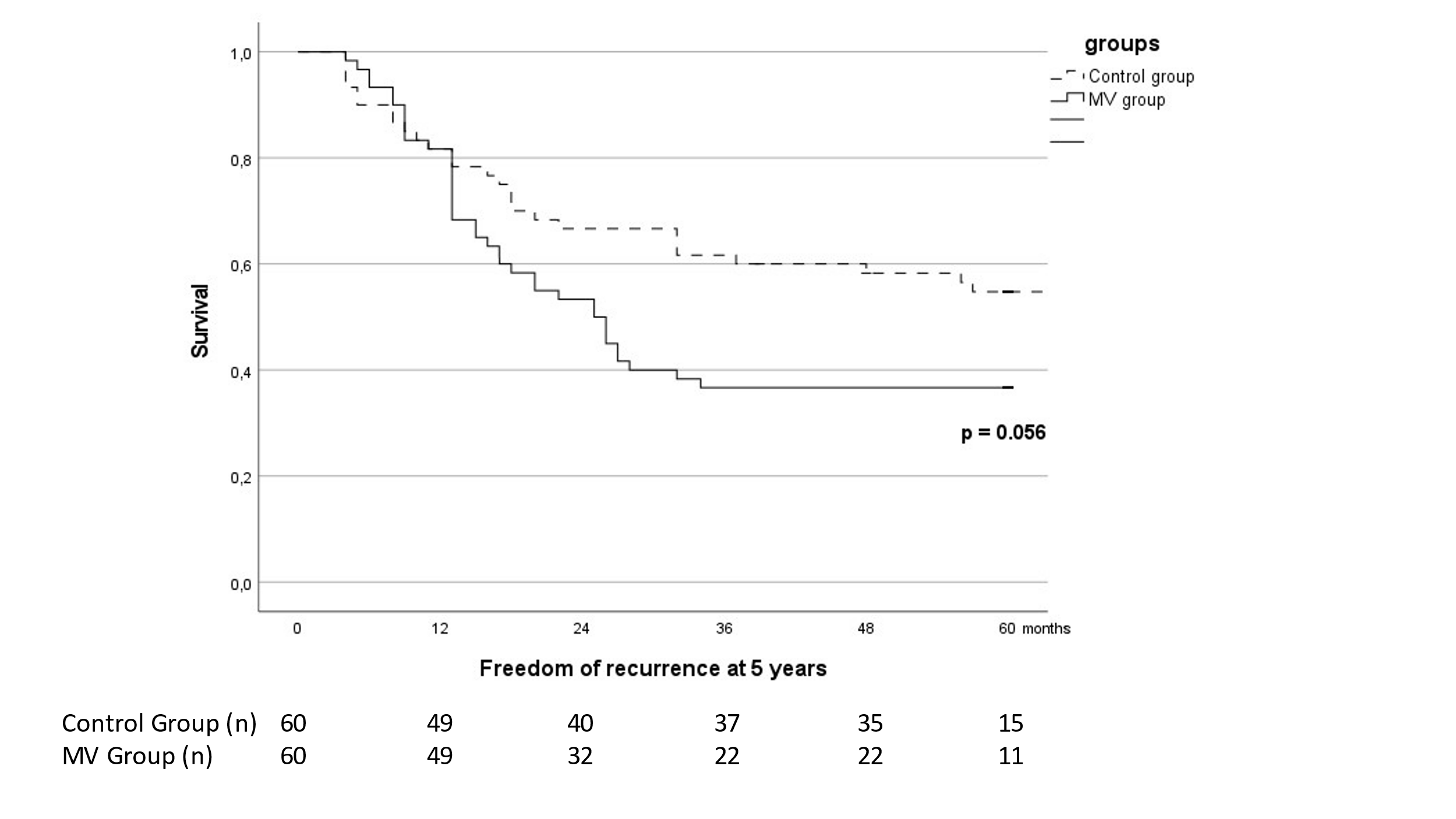


# Legends

Arrhythmia-free survival at 1-, 2- and 5-years after catheter ablation for atrial fibrillation in patients with previous mitral valve (MV) surgery patients vs control. Number of patiens at risk at each time interval is shown below the table. The p value reflects the Log-rank significance at the end of follow-up.
